# Diagnosing streptococcal pharyngotonsillitis in children and adolescents: the limitations of the clinical features☆

**DOI:** 10.1016/j.rpped.2014.11.013

**Published:** 2014-12

**Authors:** Saulo Duarte Passos

**Affiliations:** Faculdade de Medicina de Jundiaí (FMJ), Jundiaí, SP, Brazil

Barbosa Junior *et al*, address in this issue the experience of the group on a relevant matter in pediatric practice: the diagnosis of Streptococcal Pharyngotonsillitis (SPT). "Sore throat" symptoms, including pharyngitis, are common reasons for seeking medical assistance in primary care and emergency services,^1^ accounting for 12 million consultations a year in the United States in 2006, with a significant impact to the healthcare system.^2^ Although most cases of pharyngitis have viral etiology (50-80%), bacterial agents are implicated in approximately 5-30% of the cases.^3^^-^ 5 In cases of bacterial pharyngitis, infections caused by *Streptococcus pyogenes* (SGA) are the most frequent. The importance of identifying and treating them with appropriate antibiotics, both in the choice of drug and the beginning and time of administration are essential, as they reduce clinical symptoms and transmission to close contacts.^6^

The concern with diagnostic accuracy is a long-standing fact. In 1961, Stillerman & Bernstein^7 ^warned: "If you are comfortable selecting which patients with pharyngitis should receive 10 days of treatment with penicillin, you may not understand the situation." This discomfort persists even today, as there is no clinical evidence that cases of bacterial pharyngitis are more serious than viral ones or that there are differences in duration in both cases. Given these several etiological possibilities, accurate clinical diagnosis becomes a challenge, efficiently met by the study authors. Some aspects should be emphasized. Different from other studies,^7^^,^ 8 the exclusion criteria of patients due to previous antibiotic use was extended to up to 30 days for those who used Benzathine penicillin, for patients treated in emergency rooms and outpatient clinics. 

The case definition used was laboratory-based and stringent, using the combination of the results of rapid antigen detection test and / or culture for SGA. Admission criteria were broad, including upper airway symptoms, which may have interfered with the lower positivity (23.4%) of laboratory tests for SPT detection,^9^^,^ 10 when compared to other works in our country.^8^^,^ 11 The exclusion of respiratory symptoms increases the pretest prevalence of streptococcal pharyngitis. The prevalence of SPT similar to the study was found in an international multicenter study that included Brazil, pointing out to the use of the rapid test for diagnosis.^12^

The clinical characteristics presented in this study showed increased sensitivity, with low specificity for the diagnosis of SPT by SGA. These criteria could delay antibiotic treatment aimed at eradication of streptococci from the oropharynx. Antibiotics can probably prevent complications, although these are rare.^13^

The introduction of the score system resulted in higher specificity and lower sensitivity of the clinical picture. The search for suitable clinical scores has been a concern to institute SGA control policies. In 1981, Centor et al.^10^ developed criteria to predict the likelihood of SGA presence in cultures of adult patients, but it became a tool for the assessment of SG absence. Other studies with children established clinical scores aiming at an empirical antibiotic therapy, with good results.^14^^,^ 15

The clinical score is not used as a consensus, as some studies have shown that empiric treatment can lead to a significant amount of unnecessary antibiotic use^16^^,^ 18 and others have shown that there is no advantage in using clinical scores alone on the association with the rapid test.^19^ Other studies have associated other tests, such as leukogram and C-reactive protein, to clinical criteria, with improved specificity.^1 ^This subject offers a wide opportunity for studies to better understand and diagnose SGA, aiming to optimize the prescription and reduce the resistance of antibiotic agents. 

## References

[1] Alper Z, Uncu Y, Akalin H, Ercan I, Sinirtas M, Bilgel NG (2013). Diagnosis of acute tonsillopharyngitis in primary care:
a new approach for low-resource settings. J Chemother.

[2] Schappert SM, Rechtsteiner EA, Division of Health Care Statistics (2007). Ambulatory medical care utilization estimates for
2007. Vital Health Stat.

[3] Bisno AL (1996). Acute pharyngitis: etiology and
diagnosis. Pediatrics.

[4] Scottish Intercollegiate Guidelines Network (2010). Management of sore throat and indications for
tonsillectomy.

[5] Ebell MH, Smith MA, Barry HC, Ives K, Carey M (2000). The rational clinical examination. Does this patient
have strep throat?. JAMA.

[6] Lindbaek M, Francis N, Cannings-John R, Butler CC, Hjortdahl P (2006). Clinical course of suspected viral sore throat in young
adults: cohort study. Scand J Prim Health Care.

[7] Stillerman M, Bernstein S (1961). Streptococcal Pharyngitis: Evaluation of clinical
syndromes in diagnosis. Am J Dis Child.

[8] Cardoso D M, Gilio AE, Hsin SH, Machado BM, Paulis M, Lotufo JP (2013). Impact of the rapid antigen detection test in diagnosis
and treatment od acute pharyngotonsillits in a Pediatric emergency
room. Rev Paul Pediatr.

[9] Gieseker KE, Roe MH, MacKenzie T, Todd JK (2003). Evaluating the american academy of pediatrics diagnostic
standard for streptococcus pyogenes pharyngitis: backup culture versus repeat
rapid antigen testing. Pediatrics.

[10] Centor RM, Witherspoon JM, Dalton HP, Brody CE, Link K (1981). The diagnosis of strep throat in adults in the emergency
room. Med Decis Making.

[11] dos Santos AG, Berezin EN (2005). Comparative analysis of clinical and laboratory methods
for diagnosing streptococcal sore throat. J Pediatr (Rio J).

[12] Rimoin AW, Walker CL, Hamza HS, Elminawi N, Ghafar HA, Vince A (2010). The utility of rapid antigen detection testing for the
diagnosis of streptococcal pharyngitis in low-resource settings. Int J Infect Dis.

[13] Del Mar CB, Glasziou PP, Spinks AB (2004). Antibiotics for sore throat. Cochrane Database Syst Rev.

[14] Joachim L, Campos D, Smeesters PR (2010). Pragmatic scoring system for pharyngitis in low-resource
settings. Pediatrics.

[15] Roggen I, van Berlaer G, Gordts F, Pierard D, Hubloue I (2013). Centor criteria in children in a paediatric emergency
department: for what it is worth. BMJ Open.

[16] McIsaac WJ, Kellner JD, Aufricht P, Vanjaka A, Low DE (2004). Empirical validation of guidelines for the management of
pharyngitis in children and adults. JAMA.

[17] Bisno AL, Peter GS, Kaplan EL (2002). Diagnosis of strep throat in adults: are clinical
criteria really good enough?. Clin Infect Dis.

[18] McIsaac WJ, Goel V, To T, Low DE (2000). The validity of a sore throat score in family
practice. CMAJ.

[19] Little P, Hobbs FD, Moore M, Mant D, Williamson I, McNulty C (2013). Clinical score and rapid antigen detection test to guide
antibiotic use for sore throats: randomised controlled trial of PRISM (primary
care streptococcal management). BMJ.

