# Supplementary material for: Diagnosing streptococcal pharyngotonsillitis in children and adolescents: the limitations of the clinical features
Source: Rev Paul Pediatr. 2014 Dec;32(4):283–4. doi: 10.1016/j.rpped.2014.11.013 (PMC4311779; doi:10.1016/j.rpped.2014.11.013)
Supplement: Translation

# Translation: Diagnóstico da faringoamigdalite estreptocócica em crianças e adolescentes: limitações do quadro clínico☆

**Authors:** Saulo Duarte Passos

**Affiliations:** Faculdade de Medicina de Jundiaí (FMJ), Jundiaí, SP, Brasil

Barbosa Junior *et al* abordam nesta edição a experiência do grupo sobre um tema de relevância na prática pediátrica: o diagnóstico da faringoamigdalite estreptocócica (FAA). Os sintomas "dor de garganta", incluindo as faringites, são causas comuns de procura do atendimento médico nos serviços de atenção primária e de urgência,^1^ sendo responsáveis nos Estados Unidos por 12 milhões de atendimentos ao ano, em 2006, com importante impacto para o sistema de saúde.^2^ Embora a maioria dos casos de faringites seja de etiologia viral (50-80%), as bactérias estão implicadas em cerca de 5-30% dos casos.^3^^-^ 5 Nas faringites bacterianas, as infecções causadas pelo *Streptococcus pyogenes *(SGA) são as mais frequentes. A importância de identificar e tratar com antibiótico adequado, tanto na escolha do fármaco como no início e tempo de administração, são fundamentais, pois reduzem os sintomas clínicos e a transmissão para os contatos íntimos.^6^

A preocupação com a precisão diagnóstica é antiga. Em 1961, Stillerman & Bernstein^7^ alertaram: "Se você se sente à vontade para selecionar quais pacientes com faringite devem receber 10 dias de tratamento com penicilina, talvez não entenda a situação." Este desconforto persiste até os dias atuais, pois não existem evidências clínicas de que faringites bacterianas sejam mais graves do que virais ou que existam diferenças quanto à duração em ambos os casos. Diante dessas inúmeras possibilidades etiológicas, o diagnóstico clínico acurado se torna um desafio, enfrentado de forma eficiente pelos autores deste estudo. Alguns aspectos devem ser salientados. Diferente de outros estudos,^7^^,^ 8 o critério de exclusão de pacientes pelo uso prévio de antibiótico foi ampliado para até 30 dias no caso dos que usaram Penicilina Benzatina para os pacientes atendidos nas unidades de emergência e ambulatórios.

A definição de caso utilizada foi laboratorial, criteriosa, utilizando a combinação do resultado do teste rápido de detecção de antígenos e/ou cultura para SGA. Os critérios de admissão foram amplos, incluindo sintomas das vias aéreas superiores, o que pode ter intervindo na menor positividade (23,4%) dos testes laboratoriais para detecção da FAA^9^^,^ 10 quando comparados a outros trabalhos em nosso meio.^8^^,^ 11 A exclusão de sintomas respiratórios aumenta a prevalência pré-teste das faringites estreptocócicas. A prevalência de FAA semelhante à pesquisa foi encontrada num estudo multicêntrico internacional, que incluiu o Brasil, apontando para a utilização do teste rápido para diagnóstico.^12^

As características clínicas apresentadas nesta pesquisa mostraram maior sensibilidade, com baixa especificidade para o diagnóstico de FAA por SGA. Esses critérios poderiam postergar o tratamento com antibiótico visando à erradicação do estreptococo da orofaringe. Os antibióticos provavelmente podem prevenir complicações, muito embora estas sejam raras.^13^

A introdução do escore de pontos resultou maior especificidade e menor sensibilidade do quadro clínico. A procura de escores clínicos adequados tem sido uma preocupação para se instituir políticas no controle do SGA. Em 1981, Centor *et al* 10 desenvolveram critérios para prever a probabilidade da presença de SGA em culturas de pacientes adultos, mas tornou-se uma ferramenta para avaliar a ausência de SGA. Outros estudos com crianças estabeleceram escores clínicos visando uma terapia empírica antibiótica, com bons resultados.^14^^,^ 15

A pontuação clínica não é utilizada como consenso, pois alguns estudos mostram que o tratamento empírico pode levar a uma quantidade significativa de antibiótico desnecessário,^16^^-^ 18 e outros demonstram que não existe vantagem do uso escore clínico sozinho sobre a associação com teste rápido.^19^ Outras pesquisas associaram aos critérios clínicos outros exames como Leucograma e a Proteína C-reativa, com melhora da especificidade.^1^ Esse assunto oferece uma vasta oportunidade de estudos para melhores entendimento e diagnóstico do SGA, visando otimizar a prescrição e reduzir a resistência aos antibióticos.
